# Predictors of Decompressive Craniectomy in Patients with Acute Subdural Hematoma: A Retrospective Study

**Published:** 2026-09-08

**Authors:** RYOGO IKEMURA, NAOHIDE FUJITA, YASUAKI NAKAO, TAKUJI YAMAMOTO, AKIHIDE KONDO

**Affiliations:** 1 Department of Neurosurgery, Juntendo University Shizuoka Hospital, Shizuoka, Japan; 1 Department of Neurosurgery, Juntendo University Shizuoka Hospital, Shizuoka, Japan; 2 Department of Neurosurgery, Juntendo University School of Medicine, Tokyo, Japan; 2 Department of Neurosurgery, Juntendo University School of Medicine, Tokyo, Japan

**Keywords:** acute subdural hematoma, cerebral contusion, decompressive craniectomy, surgical indication, predictors

## Abstract

**Purpose:**

Appropriate indications for decompressive craniectomy (DC) in patients with acute subdural hematoma (ASDH) remain controversial. This study aimed to identify clinical factors associated with postoperative brain swelling after hematoma evacuation and to explore predictors that may support surgical decision-making regarding the need for DC.

**Materials and Methods:**

This retrospective study included 74 patients with ASDH who underwent hematoma evacuation via craniotomy between 2017 and 2021. Patients were classified into swelling and non-swelling groups according to postoperative clinical and radiological findings. Clinical, radiological, and treatment-related variables were compared using univariate and multivariate analysis. Model performance was evaluated using the coefficient of determination (R²), corrected Akaike information criterion (AICc), and C-index.

**Results:**

Twenty-one patients (28.4%) were classified into the swelling group and 53 patients (71.6%) into the non-swelling group. Univariate analysis demonstrated significant associations between postoperative brain swelling and younger age, severe consciousness disturbance (Glasgow Coma Scale [GCS] 3-8), concomitant cerebral contusion, and shorter time from injury to surgery. Multivariate logistic regression identified cerebral contusion as the only independent factor associated with brain swelling (OR, 20.95; 95% confidence interval [CI], 3.81-206.51; *P* < 0.001). The predictive model showed an R² of 0.47 and a C-index of 0.90, suggesting good discriminative ability.

**Conclusions:**

Postoperative brain swelling following surgery for ASDH was strongly associated with severe traumatic brain injury, particularly in patients with concomitant cerebral contusion. These findings may contribute to improved preoperative risk stratification and support surgical decision-making when considering decompressive craniectomy in high-risk patients.

## Introduction

Intracranial pressure (ICP) management using various therapeutic strategies is a cornerstone of treatment for patients with severe traumatic brain injury (TBI). Intracranial hematoma and brain swelling can cause brain herniation, leading to poor neurological outcomes. When ICP cannot be adequately controlled with conservative therapy, surgical intervention becomes an important treatment option. The initial surgical management of acute subdural hematoma (ASDH) involves evacuation of the hematoma responsible for intracranial mass effect. In clinical practice, decompressive craniectomy (DC), often performed in addition to hematoma evacuation, is an important surgical option for patients with marked brain swelling or cerebral contusion^1-4)^. However, few high-quality clinical studies have directly compared surgical strategies for achieving optimal ICP control. Furthermore, DC-related complications, including sinking skin flap syndrome and infection after cranioplasty, are not uncommon even among patients who survive the acute phase^5, 6)^, highlighting the need for appropriate criteria to optimize patient selection.

Evidence supporting DC has accumulated, including studies with relatively high levels of evidence such as RESCUEicp (Randomized Evaluation of Surgery with Craniectomy for Uncontrollable Elevation of Intracranial Pressure) and DECRA (Decompressive Craniectomy in Patients with Severe Traumatic Brain Injury)^7, 8)^. The Brain Trauma Foundation Guidelines for the Management of Severe TBI, 4th Edition, recommend DC with a level IIA recommendation^9)^, and Japanese guidelines also support its efficacy (*recommendation; grade B*)^10)^. However, strict indication criteria for DC have not been established. In clinical practice, surgical indications are often determined based not only on objective indices such as level of consciousness and hematoma volume, but also on institution-specific criteria and the neurosurgeon’s experience, including qualitative assessments of cerebral contusion severity and concomitant cerebral ischemia^4, 5)^.

The present study retrospectively evaluated clinical factors associated with postoperative brain swelling in patients who underwent conventional craniotomy for ASDH, with the aim of identifying clinical indicators that may support decision-making regarding the appropriate use of decompressive craniectomy in severe traumatic brain injury.

## Materials and Methods

### Patient population

This retrospective study included 74 patients who underwent hematoma evacuation via craniotomy for ASDH at Juntendo University Shizuoka Hospital between January 1, 2017, and December 31, 2021. Patients younger than 18 years, those with ASDH at atypical locations, and those with coexisting active diseases under treatment were excluded. As this study was a retrospective observational study, the sample size was determined by the number of eligible patients during the study period, rather than by a priori power calculation. All consecutive eligible patients were included. The characteristics of the overall cohort and those stratified by DC status are summarized in Table 1. This study was conducted in accordance with the ethical principles of the Declaration of Helsinki and approved by the Juntendo University Ethics Committee (Approval No. E24-0076-S01; approval date: May 27, 2024). The requirement for informed consent was waived because of the retrospective study design and the use of anonymized data.

**Table 1:** Baseline characteristics stratified by decompressive craniectomy status

Variable	Overall (n = 74)	Clinical procedure		*P* value
		Primary DC (n = 28)	Non-primary DC (n = 46)	
Demographics				
Age, years, median (IQR)	80 (72-85)	82 (76-86)	77 (66-81)	*P* = 0.006
Age < 65 years, n (%)	10 (13.5%)	7 (25.0%)	3 (6.5%)	*P* = 0.042
Sex, male/female	48 / 26	18/10	30 / 16	*P* = 1.000
Clinical severity				
GCS 3-8, n (%)	31 (41.9%)	17 (60.7%)	14 (30.4%)	*P* = 0.029
Anisocoria, n (%)	33 (44.6%)	17 (60.7%)	16 (34.8%)	*P* = 0.060
Talk and deterioration, n (%)	31 (41.9%)	14 (50.0%)	17 (37.0%)	*P* = 0.470
Radiological findings				
Hematoma side (Lt./Rt.)	39 / 35	18 / 10	21 / 25	*P* = 0.097
Hematoma thickness, mm	18.9 ± 7.6	18.3 ± 8.7	19.2 ± 6.9	*P* = 0.611
Midline shift, n (%)				
< 10 mm	7 (9.5%)	9 (32.1%)	17 (37.0%)	*P* = 0.839
10-20 mm	37 (50.0%)	14 (50.0%)	22 (47.8%)	
> 20 mm	30 (40.5%)	5 (17.9%)	7 (15.2%)	
Complicated cerebral contusion, n (%)	31 (41.9%)	19 (67.9%)	12 (26.1%)	*P* = 0.002
Total contusion index	0 (0-4)	2 (0-8)	0 (0-1)	*P* = 0.004
Physiological variables				
Systolic blood pressure category, n (%)				*P* = 0.127
< 110 mmHg	5 (6.8%)	2 (7.1%)	3 (6.5%)	
110-180 mmHg	41 (55.4%)	11 (39.3%)	30 (65.2%)	
> 180 mmHg	28 (37.8%)	15 (53.6%)	13 (28.3%)	
Hyperthermia, n (%)	6 (8.1%)	2 (7.1%)	4 (8.7%)	*P* = 1.000
Hypoxia, n (%)	5 (6.8%)	4 (14.3%)	1 (2.2%)	*P* = 0.074
Treatment-related variables				
Antithrombotic therapy, n (%)	23 (31.1%)	10 (35.7%)	13 (28.3%)	*P* = 0.492
Time from injury to surgery, min	412.47 ± 322.40	316.09 ± 173.79	481.75 ± 384.42	*P* = 0.037

Values are presented as mean ± standard deviation (SD) or median (interquartile range, IQR), as appropriate. DC: decompressive craniectomy, GCS: Glasgow Coma Scale; IQR: interquartile range. *P* values were calculated using the Student’s t-test or Mann-Whitney U test for continuous variables and the chi-square test for categorical variables.

### Surgical indications, procedures, and perioperative management

Before surgery, all patients received appropriate respiratory and hemodynamic management, and osmotic therapy (e.g., mannitol) was administered when intracranial hypertension was suspected. The standard procedure consisted of appropriately sized craniotomy, hematoma evacuation, identification of the bleeding source, and meticulous hemostasis. Based on intraoperative findings, such as acute brain swelling during surgery, difficulty achieving adequate hemostasis, or obvious parenchymal brain injury, decompressive craniectomy (DC) with expansive duroplasty using artificial dura was performed at the discretion of the attending neurosurgeon. Postoperatively, ICP was managed in the intensive care unit with continuous ICP monitoring. Patients with severe TBI received comprehensive care, including respiratory and hemodynamic support and pharmacological therapy. In patients managed primarily with ICP monitoring, secondary DC was considered when ICP exceeded 25 mmHg for more than 30 minutes.

For this study, brain swelling was defined as early radiological signs of cerebral herniation: compression or obliteration of the basal cisterns, midline shift > 5 mm, extracranial herniation of brain parenchyma > 10 mm, or diffuse effacement of the cerebral sulci.

Cranioplasty using autologous bone was performed after normalization of ICP, typically ≥ 3 weeks after the initial surgery. Temporary ventricular drainage or permanent cerebrospinal fluid shunting was performed in patients who developed hydrocephalus. The study followed a normothermia-based management protocol and excluded patients treated with therapeutic hypothermia or barbiturate therapy.

### Clinical parameters, definitions, and outcomes

The following variables were retrospectively evaluated: age, sex, level of consciousness (Glasgow Coma Scale [GCS]), “*talk and deterioration*”, hypertension, hyperthermia, hypoxia, anisocoria, hematoma side, preoperative hematoma thickness, type of parenchymal contusion, degree of midline shift, antithrombotic drug use, and time from injury to surgery (defined as the interval from injury onset to skin incision).

Elderly patients were defined as those aged ≥ 65 years. Severe consciousness disturbance was defined as GCS 3-8 and mild-to-moderate disturbance as GCS 9-15. Systolic blood pressure at presentation was categorized as < 110 mmHg, 110-180 mmHg, or > 180 mmHg. Hyperthermia was defined as body temperature ≥ 38°C and hypoxia as percutaneous oxygen saturation < 90%.

Hematoma volume was calculated using the ABC method^11)^. Hematomas were classified as simple or complicated according to the presence of parenchymal brain injury^12)^. Cerebral contusion severity was quantified using the Adams’ Regional Contusion Index (total contusion index, 0-72)^13)^.

Clinical outcomes were assessed using the Glasgow Outcome Scale (GOS) at 30 days (1-3, poor outcome; 4-5, good outcome). Continuous variables are presented as mean ± standard deviation or median (interquartile range), as appropriate. A two-sided P value < 0.05 was considered statistically significant.

### Group classification

Patients were classified into four groups according to primary DC and the presence of postoperative brain swelling (Table 2). For secondary analyses, Groups I and III were combined as the swelling group, and Groups II and IV as the non-swelling group based on clinical and imaging findings. Group assignment was determined by neurosurgeons blinded to the study outcomes. The classification algorithm is shown in Figure 1.

**Table 2:** Group classification based on decompressive craniectomy and postoperative brain swelling

Group	Primary DC	Postoperative brain swelling	Clinical description
I	Yes	Yes	Primary DC with postoperative brain swelling
II	Yes	No	Primary DC without postoperative brain swelling
III	No	Yes	No primary DC with brain swelling (including secondary DC or fatal swelling)
IV	No	No	No DC without brain swelling
DC: decompressive craniectomy The definition of brain swelling: compression of the basal cisterns, midline shift > 5 mm, extracranial herniation of brain parenchyma > 10 mm, or diffuse effacement of the cerebral sulci.			
Secondary analysis classification		
Swelling group:		Group I + Group III
Non-swelling group:		Group II + Group IV

**Figure 1:**
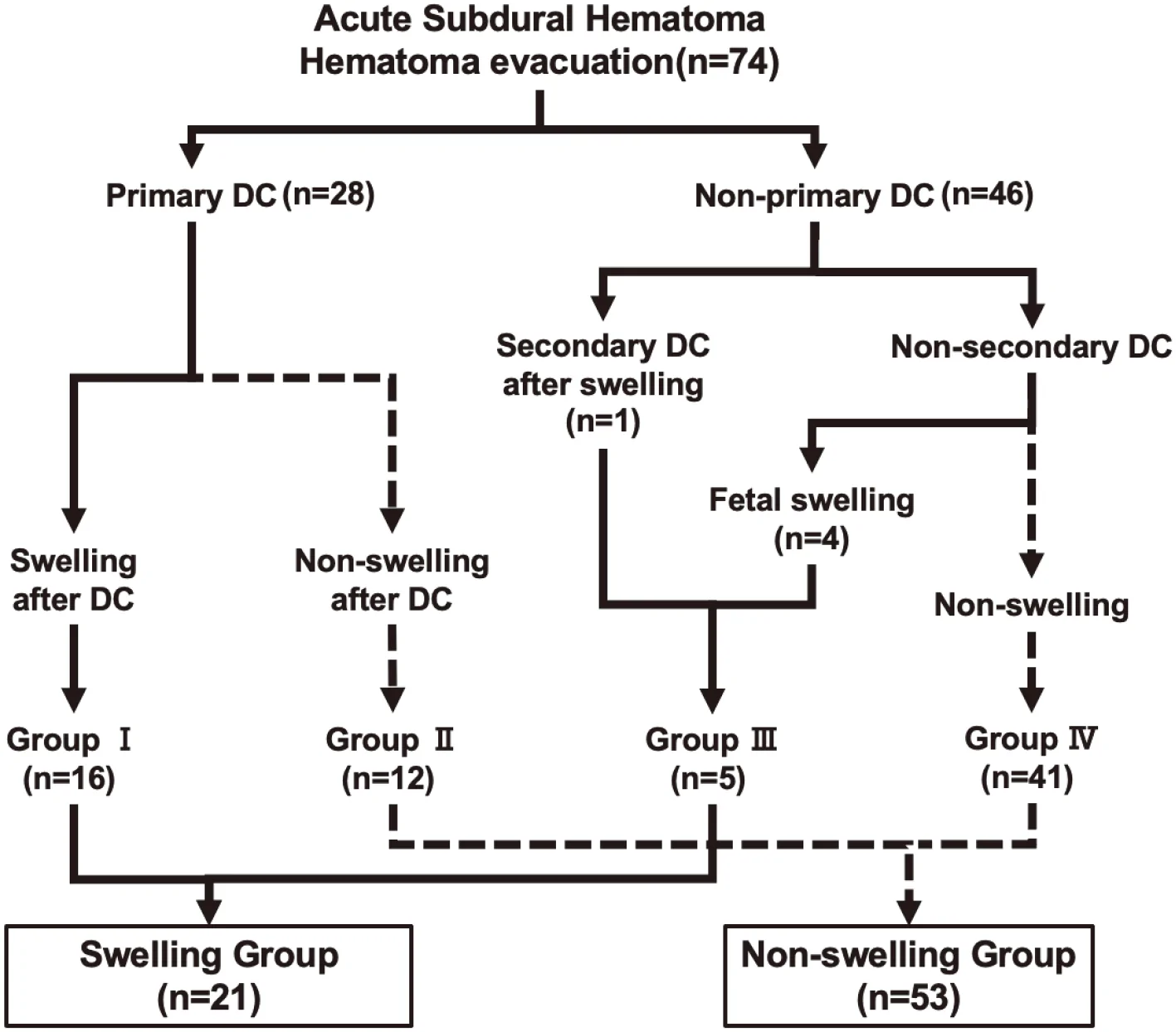
Secondary DC was considered when ICP > 25 mmHg for more than 30 minutes. Group classification algorithm. Patients undergoing primary decompressive craniectomy (DC) judged as effective were defined as Group Ⅰ, and not necessary as Group II. Patients requiring secondary DC were defined as Group III, and not requiring any additional treatment as Group IV.

### Statistical analysis

Differences in continuous variables between the swelling and non-swelling groups were assessed using the Student’s *t*-test or Mann-Whitney *U* test, as appropriate. The time from injury to surgery, which was significant in the *t*-test, was dichotomized using an exploratory cutoff value determined from receiver operating characteristic (ROC) curve analysis. Categorical variables were compared using the chi-square test.

Variables significant in univariate analysis were entered into a multivariable logistic regression model to identify independent factors associated with postoperative brain swelling. Model performance was evaluated using the coefficient of determination (R^2^), the corrected Akaike information criterion (AICc), and the c-statistic. Results are presented as odds ratios (OR) with 95% confidence intervals (CI) and *P* values.

All analyses were performed using JMP^®^ Pro 18.0 (SAS Institute Inc., Cary, NC, USA). All tests were two-sided, and *P* < 0.05 was considered statistically significant.

## Results

The baseline characteristics of the overall cohort are summarized in Table 1. The study cohort comprised 74 patients with a median age of 80 years (interquartile range [IQR], 72-85), of whom 26 (35.1%) were female.

According to the predefined classification algorithm (Table 2 and Figure 1), patients were categorized into swelling and non-swelling groups. Twenty-one patients (28.4%; male : female = 13 : 8) were classified into the swelling group and 53 patients (71.6%; male : female = 35 : 18) into the non-swelling group.

Clinical parameters for each group are presented in Table 3. The median age was 75 years (IQR, 58-80) in the swelling group and 82 years (IQR, 75-86) in the non-swelling group, with the latter being significantly older (*P* < 0.001).

**Table 3:** Univariate analysis of factors associated with brain swelling

Variable	Swelling (n = 21)	Non-swelling (n = 53)	OR (95% CI)	*P* value
Demographics				
Age, years, median (IQR)	75 (58-80)	82 (75-86)		*P* < 0.001
Age < 65 years, n (%)	8 (38.1%)	2 (3.8%)	0.06 (0.01-0.34)	*P* < 0.001
Sex, male/female	13/8	35/18	1.20 (0.42-3.41)	*P* = 0.790
Clinical severity				
GCS 3-8, n (%)	15 (71.4%)	16 (30.2%)	5.78 (1.90-17.61)	*P* = 0.002
Anisocoria, n (%)	13 (61.9%)	20 (37.7%)	0.37 (0.13-1.06)	*P* = 0.073
Talk and deterioration, n (%)	7 (33.3%)	24 (45.3%)	1.66 (0.58-4.76)	*P* = 0.437
Radiological findings				
Hematoma side (Lt./Rt.)	14/7	25/28	0.45 (0.16-1.28)	*P* = 0.196
Hematoma thickness, n(%)				*P* = 0.358
< 10 mm	3 (14.3%)	4 (7.5%)		
10-20 mm	12 (57.1%)	25 (47.2%)		
> 20 mm	6 (28.6%)	24 (45.3%)		
Midline shift, n(%)				*P* = 0.438
< 10 mm	5 (23.8%)	21 (39.6%)		
10-20 mm	12 (57.1%)	24 (45.3%)		
> 20 mm	4 (19.1%)	8 (15.1%)		
Complicated cerebral contusion, n (%)	17 (81.0%)	14 (26.4%)	11.84 (3.40-41.27)	*P* < 0.001
TCI ≥ 3	12 (57.1%)	12 (22.6%)	4.56 (1.55-13.38)	*P* = 0.006
Physiological variables				
Systolic blood pressure category, n (%)				*P* = 0.385
< 110 mmHg	2 (9.5%)	3 (5.6%)		
110-180 mmHg	9 (42.9%)	32 (60.4%)		
> 180 mmHg	10 (47.6%)	18 (34.0%)		
Hyperthermia, n (%)	0 (0%)	6 (11.3%)		
Hypoxia, n (%)	1 (4.8%)	4 (7.5%)	1.63 (0.17-15.5)	*P* = 1.000
Treatment-related variables				
Antithrombotic therapy, n (%)	4 (19.0%)	19 (35.8%)	2.38 (0.70-8.09)	*P* = 0.265
Time from injury to surgery < 350 min	16 (84.2%)	15 (41.7%)	0.13 (0.03-0.54)	*P* = 0.004

Odds ratios (OR) were calculated using univariate logistic regression analysis. Nineteen cases with no description about surgical time were not included in this analysis. CI: confidence interval; CT: Computed tomography; GCS: Glasgow Coma Scale; IQR: Interquartile range OR: odds ratio; SD: Standard deviation; TCI: total contusion index

Univariate analysis demonstrated that brain swelling was significantly associated with age < 65 years (odds ratio [OR], 0.060; 95% confidence interval [CI], 0.01-0.34; *P* < 0.001), GCS 3-8 (OR, 5.78; 95% CI, 1.90-17.61; *P* = 0.002), and concomitant cerebral contusion (OR, 11.84; 95% CI, 3.40-41.27; *P* < 0.001). Cerebral contusion severity was assessed using the Adams’ Regional Contusion Index, and a total contusion index ≥ 3 was also significantly associated with brain swelling (OR, 4.56; 95% CI, 1.55-13.38; *P* = 0.006).

Time from injury onset was clearly documented in 55 of the 74 patients; cases without exact time records were excluded from this analysis. The mean time from injury to surgery was significantly shorter in the swelling group (280.1 ± 120.5 min) than in the non-swelling group (482.4 ± 366.2 min) (*P* = 0.007). Exploratory receiver operating characteristic (ROC) curve analysis identified a cutoff value of 351 minutes (sensitivity 58.3%, specificity 84.2%). The area under the ROC curve (AUC) was 0.66 (95% CI, 0.47-0.85). Based on this cutoff, patients were dichotomized into < 350 minutes and ≥ 350 minutes groups, which showed a significant association with brain swelling (OR, 0.13; 95% CI, 0.03-0.54; *P* = 0.004).

No significant differences were observed for sex, “talk and deterioration,” vital sign abnormalities, anisocoria, hematoma side, hematoma thickness, midline shift, or antithrombotic medication use. However, anisocoria showed a tendency toward association with brain swelling (OR, 0.37; 95% CI, 0.13-1.06; *P* = 0.073). All patients in the swelling group had poor outcomes at 30 days (*P* = 0.0001).

Variables with *P* < 0.05 in univariate analysis were entered into multivariate logistic regression analysis. Multivariate analysis showed cerebral contusion as the only independent factor associated with brain swelling (OR, 20.95; 95% CI, 3.81-206.51; *P* < 0.001), whereas age, GCS, and time from injury to surgery were not significant. The model showed a coefficient of determination (R^2^) of 0.47, indicating that approximately 47% of the variance associated with brain swelling was accounted for by the model. Model selection using the corrected Akaike Information Criterion yielded an AICc of 48.93. The C-index was 0.90, suggesting good discriminative ability of the model. Multivariate analysis results are presented in Table 4.

**Table 4:** Multivariate logistic regression analysis for predictors of brain swelling

Variable	Adjusted OR	95% CI	*P* value
Age < 65 years	11.04	0.91-348.63	*P* = 0.060
GCS 3-8	4.63	0.78-38.43	*P* = 0.092
Complicated cerebral contusion	20.95	3.81-206.51	*P* < 0.001
Time from injury to surgery < 350 min	3.08	0.56-19.91	*P* = 0.195

GCS: Glasgow Coma Scale, CI: confidence interval, OR: odds ratio Variables with *P* < 0.05 in univariate analysis were entered into the multivariate model. Due to limited event numbers, model stability should be interpreted with caution.

Characteristics of the five patients who did not underwent primary DC and were later classified in Group III due to brain swelling are shown in Table 5. Four of the five patients had severe traumatic brain injury with GCS score of 8 or less, and all patients had concomitant cerebral contusion, although the severity varied among cases. In contrast, no consistent pattern was observed regarding the time from injury to surgery.

**Table 5:** Characteristics of the 5 cases in Group III

Age	Sex	GCS	Contusion type	Time from injury to Surgery
84	male	≥ 9	complicated	≥ 350 min
77	male	3-8	simple	≥ 350 min
58	male	3-8	simple	unknown
47	male	3-8	complicated	< 350 min
79	female	3-8	complicated	< 350 min

## Discussion

This retrospective study evaluated patients with ASDH who underwent craniotomy for hematoma evacuation, with or without decompressive craniectomy (DC), to identify factors associated with postoperative brain swelling that may help guide decision-making regarding decompressive craniectomy. ASDH is caused by intracranial hypertension due to hematoma mass effect and focal injury such as cerebral contusion^14-16)^. The molecular mechanisms underlying poor outcomes remain complex^17, 18)^, making treatment decisions challenging.

Normalization of ICP remains a fundamental therapeutic strategy, second only to circulatory and respiratory stabilization. When conservative measures such as hyperventilation or osmotic therapy fail, surgical decompression—including DC or removal of intraparenchymal mass lesions—becomes necessary. However, predicting the need for additional decompression based solely on preoperative findings is often difficult and may result in delayed or unnecessary intervention.

Younger patients were more likely to develop postoperative brain swelling, suggesting that careful consideration of DC may be warranted in such high-risk patients. Age-related brain atrophy in elderly patients may increase ICP buffering capacity through enlargement of the cerebrospinal fluid space^6, 19-21)^, thereby reducing the need for DC. In contrast, lower intracranial compliance in younger patients^22)^ predisposes them to rapid ICP elevation and a higher likelihood of requiring DC. Poor preoperative level of consciousness was also associated with increased DC requirement, likely reflecting greater injury severity, consistent with previous findings^23)^.

Early surgery has been associated with improved outcomes in ASDH, particularly when performed within 2-4 hours after injury^24, 25)^. Accordingly, patients meeting surgical criteria should undergo prompt treatment^26)^. In this study, time from injury to surgery was significantly shorter in the swelling group, and DC was more frequently required in patients operated on within 350 minutes (*P* = 0.004). This likely reflects confounding by injury severity, as more severe cases undergo earlier surgery and are also more likely to require DC.

Although surgical treatment for cerebral contusions associated with ASDH is established^27, 28)^, clear criteria for DC remain undefined. A TCI ≥ 3 was significantly associated with DC requirement, suggesting that the presence of contusion itself may influence DC indication more than its absolute severity. In addition, delayed deterioration, including “talk and deteriorate” phenomena, underscores the importance of careful preoperative evaluation and consideration of proactive DC in high-risk patients.

No significant association was observed between DC and traditional markers of herniation such as pupillary abnormalities or midline shift. Pupillary irregularities caused by cerebral ischemia may not always correlate with ICP elevation^29)^, suggesting that continuous ICP monitoring is valuable. Conversely, localized brainstem compression may adversely affect prognosis even without global ICP elevation, highlighting the need to integrate ICP monitoring with detailed imaging assessment.

The RESCUE-ASDH randomized trial reported comparable long-term disability and quality-of-life outcomes between craniotomy and DC^30)^. However, it did not evaluate predictors of DC necessity. Previous studies^2, 4)^ have examined factors related to DC requirement without definitive conclusions. The present findings may assist in refining preoperative decision-making regarding consideration of DC. Interestingly, re-examination of the five patients classified in Group III showed that four patients had severe consciousness disturbance (GCS ≤ 8), and all patients had concomitant cerebral contusion. Although statistical analysis was not feasible because of the limited number of cases, these findings suggest that some of the risk factors identified in the present model were also present in patients who subsequently developed postoperative brain swelling despite not undergoing primary DC.

## Limitations

This single-center retrospective study is subject to inherent selection bias. Although DC decisions were made by board-certified neurosurgeons, the influence of clinical judgment cannot be excluded, particularly in primary DC cases.

The DC group may have included patients in whom early DC prevented subsequent swelling, whereas some non-DC cases later required secondary DC. The swelling group included 21 events while four predictors were entered into the multivariable model, raising concern for overfitting and instability of estimates.

Cerebral contusion was shown as an independent factor but is intrinsically related to surgical indication and injury severity, limiting outcome comparisons between groups. Furthermore, the 95% confidence interval for cerebral contusions is wide; this may be due to the limited number of cases or potential bias resulting from insufficient data. Therefore, caution is required when interpreting the results.

Functional outcome was assessed at 30 days using GOS, which may underestimate later recovery. Finally, the AUC of 0.66 indicates modest discriminative performance of the TCI cutoff and warrants cautious clinical interpretation.

## Conclusions

This retrospective study identified several factors associated with postoperative brain swelling in patients with ASDH, including younger age, severe impairment of consciousness, concomitant cerebral contusion, and shorter time from injury to surgery. Although these findings do not establish definitive criteria for DC, they may contribute to improved preoperative risk stratification and support surgical decision-making when considering DC in high-risk patients.

## Author contributions

RI, YN and TY designed and conceived this study. RI and NF collected data. RI and TY analyzed and interpreted the results and drafted the manuscript. AK supported statistical analyses. All authors read and approved the final manuscript.

## Conflicts of interest statement

The authors declare that there are no conflicts of interest.
